# The GOBLET training portal: a global repository of bioinformatics training materials, courses and trainers

**DOI:** 10.1093/bioinformatics/btu601

**Published:** 2014-09-04

**Authors:** Manuel Corpas, Rafael C. Jimenez, Erik Bongcam-Rudloff, Aidan Budd, Michelle D. Brazas, Pedro L. Fernandes, Bruno Gaeta, Celia van Gelder, Eija Korpelainen, Fran Lewitter, Annette McGrath, Daniel MacLean, Patricia M. Palagi, Kristian Rother, Jan Taylor, Allegra Via, Mick Watson, Maria Victoria Schneider, Teresa K. Attwood

**Affiliations:** ^1^The Genome Analysis Centre, Norwich, ^2^ELIXIR, Wellcome Trust Genome Campus, Hinxton, UK, ^3^The Swedish University for Agricultural Sciences, Uppsala, Sweden, ^4^European Molecular Biology Laboratory, Heidelberg, Germany, ^5^Ontario Institute for Cancer Research, Toronto, Canada, ^6^Instituto Gulbenkian de Ciência, Oeiras, Portugal, ^7^The University of New South Wales, Sydney, Australia, ^8^Netherlands Bioinformatics Centre, ^9^Department of Bioinformatics, Radboud Medical Center, Nijmegen, The Netherlands, ^10^CSC - IT Center for Science Ltd., Espoo, Finland, ^11^Whitehead Institute for Biomedical Research, MIT, Cambridge, MA, USA, ^12^CSIRO, Bioinformatics Core, Canberra, Australia, ^13^The Sainsbury Laboratory, Norwich Research Park, Norwich, UK, ^14^SIB Swiss Institute of Bioinformatics, 1 Rue Michel Servet, Genève, Switzerland, ^15^Academis, Illstrasse 12, 12161 Berlin, Germany, ^16^The Nowgen Centre, 29 Grafton Street, Manchester, UK, ^17^Department of Physics, Sapienza University, Rome, Italy, ^18^The Roslin Institute, Edinburgh, UK and ^19^The University of Manchester, Manchester, UK

## Abstract

**Summary:** Rapid technological advances have led to an explosion of biomedical data in recent years. The pace of change has inspired new collaborative approaches for sharing materials and resources to help train life scientists both in the use of cutting-edge bioinformatics tools and databases and in how to analyse and interpret large datasets. A prototype platform for sharing such training resources was recently created by the Bioinformatics Training Network (BTN). Building on this work, we have created a centralized portal for sharing training materials and courses, including a catalogue of trainers and course organizers, and an announcement service for training events. For course organizers, the portal provides opportunities to promote their training events; for trainers, the portal offers an environment for sharing materials, for gaining visibility for their work and promoting their skills; for trainees, it offers a convenient one-stop shop for finding suitable training resources and identifying relevant training events and activities locally and worldwide.

**Availability and implementation:** http://mygoblet.org/training-portal

**Contact:** manuel.corpas@tgac.ac.uk

## 1 INTRODUCTION

Technologies underpinning the life sciences are constantly evolving (Abeln *et al.*, 2013) and, at the same time, are spurring development of new methods for data analysis and interpretation (Carvalho and Rustici, 2013; Brazas and Ouellette, 2013; Libeskind-Hadas and Bush, 2013). Researchers—students and professionals alike—therefore constantly need to acquire new skills to keep abreast of the latest developments (Schneider *et al.*, 2010; Via *et al.*, 2011, 2013; Vincent and Page, 2013). Attempting to address this need, the Global Organisation for Bioinformatics Learning, Education and Training (GOBLET) has established a training portal, spanning the fields of bioinformatics, biocuration, biocomputing and computational biology. The portal provides a freely available collection of materials and courses, and a catalogue of trainers, classified by tags. The tags make it easy to find and share materials and to identify trainers with appropriate expertise. The portal inherits much of the functionality of the prototype BTN website (Schneider *et al.*, 2012), extending its features to accommodate the diverse needs of global communities of life scientists: enhancements include (i) the addition of features such as the definition of fields for describing materials, to make them more discoverable, and (ii) the possibility to add course pages, linked to their associated materials, so that the portal is both a repository and a record of what is to be, and what was, taught at a given time, rather than just a bag of disconnected contents.

## 2 THE TRAINING PORTAL

The portal, built using the Drupal content management system, embodies three main entities: members, materials and courses. Members may be individuals representing their own interests, or they may represent particular organizations or groups (national and/or international networks and societies, research institutes, foundations, academic groups and so on).

**Fig. 1.**
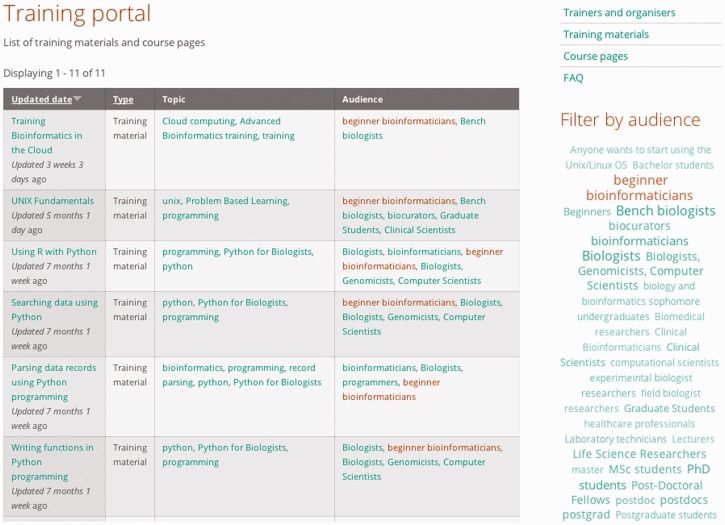
Filtering courses and materials using the audience tag ‘*beginner bioinformatician*’ retrieves 11 results. On the right-hand side, all available tags are shown, with font sizes reflecting the relevance of the matched courses and materials

Materials are available under a CC BY-SA 3.0 licence for download and use, but materials and courses may currently be uploaded only by registered members and/or by GOBLET-affiliated trainers—i.e. upload to the site requires registration with the GOBLET Foundation (this helps to minimize spurious entries and maintain quality standards). Materials may be presentations, tutorials, datasets, case studies, curricula, etc.; courses may be workshops, summer schools or road shows, and may be linked to their respective materials once uploaded to the repository. All contents are tagged: tags allow classification or filtering of entities by keyword, making them easy to find—the main content filters are currently ‘audience’ and ‘topic’. Figure 1 shows a filtered view of courses and materials using audience tag ‘*beginner bioinformatician*’. Audience tags are particularly valuable because they pinpoint the level to which specific materials are geared. In addition to filtering, a star-rating scheme can be used to rank-order search results. The portal also provides a catalogue of trainers, with information describing their fields of expertise, and lists of their training materials and courses. This registry of trainers and their learning resources may prove useful for course or event organizers, wishing to identify trainers with particular skill sets, and for students and learners looking to acquire particular skills or techniques (here, the rating system may help to guide their choice of materials). To date, 83 training materials and courses have been uploaded to the portal, which also contain profile information on 66 registered trainers and course organizers; it currently receives ∼500+ hits per day.

To augment its functionality, the portal is seamlessly integrated with the iAnn platform (Jimenez *et al.*, 2013), a distribution system for generic bioinformatics events around the world. Several future enhancements are planned: among these, we are devising an ontology to standardize the portal’s content tags. In developing this ontology, we are collaborating with initiatives like ELIXIR-UK to allow course providers to share information in a standard manner, facilitating the distribution of this information through third-party federated resources.

Overall, the portal offers an established, supported and sustainable infrastructure for individuals, groups, organizations, projects, etc., that are routinely producing training materials and courses, but have no framework for organizing or storing their content: e.g. the AllBio consortium recently saw the advantage of exploiting the portal, rather than creating yet another training resource that would be unsupported when project funds cease.

## 3 CONCLUSIONS

The GOBLET training portal is a pioneering global initiative to federate information relevant to bioinformatics, biocomputing, biocuration and computational biology trainers, courses and materials. The contents are free to download, and are catalogued according to topic and audience to enhance their discoverability. The portal is an evolving resource, whose functionality and utility will grow in harmony with the evolving needs of the global life science research communities it serves, and synergistically with bioinformatics training initiatives around the world.
